# RDS-04-010: a novel atypical DAT inhibitor that inhibits cocaine taking and seeking and itself has low abuse potential in experimental animals

**DOI:** 10.1038/s41398-025-03391-7

**Published:** 2025-05-24

**Authors:** Omar Soler-Cedeno, Ewa Galaj, Benjamin Klein, Jianjing Cao, Guo-Hua Bi, Amy Hauck Newman, Zheng-Xiong Xi

**Affiliations:** 1 Medicinal Chemistry Section, Molecular Targets and Medications Discovery Branch, National Institute on Drug Abuse Intramural Research Program, Baltimore, MD USA; 2 Department of Psychological and Brain Sciences, Colgate University, Hamilton, NY USA

**Keywords:** Addiction, Neuroscience, Pharmacology

## Abstract

Cocaine use disorder (CUD) is a severe public health problem, and currently, there is no FDA-approved medication for its treatment. Atypical dopamine (DA) transporter (DAT) inhibitors display low addictive liability by themselves and may have therapeutic potential for treatment of psychostimulant use disorders. Here, we report that RDS-04-010, a novel atypical DAT inhibitor that binds to an inward-facing conformation of DAT due to its sulfoxide moiety, displayed distinct pharmacological profiles in animal models of addiction from its sulfide analog, RDS-03-094, a DAT inhibitor that binds to a more outward-facing conformation. Systemic administration of RDS-04-010 dose-dependently inhibited cocaine self-administration, shifted the cocaine self-administration dose-response curve downward, decreased motivation for cocaine seeking under progressive-ratio reinforcement conditions, and inhibited cocaine-primed reinstatement of drug-seeking behavior. RDS-04-010 alone neither altered optical brain-stimulation reward nor evoked reinstatement of drug-seeking behavior. RDS-04-010 substitution for cocaine was not able to maintain self-administration in rats trained to self-administer cocaine. In contrast, RDS-03-094 displayed more cocaine-like reinforcing effects. Its pretreatment upward-shifted both the cocaine self-administration dose-response and optical brain-stimulation reward curves. RDS-03-094 alone was able to reinstate extinguished cocaine-seeking behavior and sustain self-administration during a substitution test. Collectively, these findings suggest that RDS-04-010 is a novel atypical DAT inhibitor with favorable therapeutic potential in reducing cocaine-taking and -seeking behavior with low addictive liability. Moreover, this extensive behavioral evaluation further confirms the role that DAT binding conformation plays in the distinctive profiles of atypical DAT inhibitors that prefer the inward facing conformation.

## Introduction

Cocaine use disorder (CUD), characterized by the compulsive use of cocaine despite its negative medical, social, and both physical and psychological consequences, affects millions globally. In the United States alone, approximately 5 million people aged 12 and above use cocaine annually, with 1.3 million meeting the DSM-5 criteria for CUD [1]. Despite extensive research over the past three decades, there is no FDA-approved medication for treating CUD.

The rewarding and addictive effects of cocaine are primarily mediated by its ability to block dopamine (DA) reuptake [2–5]. By inhibiting the reuptake of DA via the DA transporter (DAT), cocaine increases DA concentrations in the synaptic cleft, thereby enhancing DA transmission and producing potent rewarding and psychomotor-stimulating effects [6]. Notably, early studies indicate that some DAT inhibitors such as amphetamine and methylphenidate exhibit similar cocaine-like rewarding and stimulating effects [7, 8], while other DAT inhibitors, like GBR12909, benztropine, modafinil and their analogs, do not produce cocaine-like locomotor activity or self-administration [9–16]. As a result, DAT inhibitors are classified into typical (cocaine-like) and atypical (cocaine-unlike) categories.

How do DAT inhibitors that block the same transporters as cocaine produce such distinct behavioral effects? Computational modeling, molecular dynamics simulations, and most recently, cryoEM structures of the human DAT (hDAT) [17–19] have revealed critical structural confirmation that will undoubtedly help answer this complex question [12, 20–22]. Cocaine and cocaine analogs, such as β-CFT (also known as WIN25428) are potent and rewarding psychostimulants in animal models and prefer binding DAT in an outward-facing conformation, now confirmed with cryoEM structures [18]. In contrast, cryoEM structures of benztropine and GBR12909 have confirmed previously described preference for a more inward facing conformation of hDAT, using molecular pharmacology and molecular dynamics [17, 23, 24]. In addition to a library of benztropine analogues that we have described as behaviorally atypical [13, 25–27], we have more recently discovered atypical DAT inhibitors based on modafinil, such as JJC8-091, that bind to a more occluded inward-facing conformation of DAT. This binding preference has been associated with lack of cocaine-like behaviors, including reduced addictive liability and has opened the possibility of development as CUD therapeutics [9, 22, 28, 29].

Early studies indicated that GBR12909 mitigated cocaine-induced increases in extracellular DA and decreased cocaine-maintained behavior in rhesus monkeys [30–32], suggesting that this atypical DAT inhibitor may be useful for treating psychostimulant use disorders. However, further development of this drug was terminated in clinical trials due to QT prolongation, indicating possible cardiotoxicity [13, 33, 34]. Additionally, modafinil and its R-enantiomer (*R*-modafinil), FDA-approved medications for the treatment of narcolepsy, have also been explored in clinical trials for the treatment of CUD. However, results have been mixed – some studies showed a reduction in cocaine use, while others did not [35–37].

Over the past decade, we and others have modified modafinil’s structure, generating a several series of atypical DAT inhibitors with improved pharmacological and physicochemical profiles [7, 28, 38–44]. Our early studies yielded two lead compounds: JJC8–088 and JJC8–091 [28]. Preclinical studies indicate that JJC8-088 is cocaine-like in rodent behavioral tests, while JJC8-091 is an atypical DAT inhibitor that reduces cocaine self-administration and blocks reinstatement of cocaine-seeking behavior [29]. JJC8-091 itself does not appear to have addictive liability, as it neither sustains self-administration in cocaine substitution tests nor produces reinstatement of drug-seeking behavior in rats [29], supporting its therapeutic potential in treating CUD. These data were supported by additional studies indicating that JJC8-091 is also effective in reducing short- and long-access methamphetamine self-administration in rats [45]. However, mixed results were observed in non-human primates after chronic administration of JJC8-091 [46]. Nevertheless, JJC8-091 continued to distinguish itself from the addictive liability of the typical DAT inhibitor, JJC8-088, as recently demonstrated in cocaine-experienced rhesus monkeys wherein it was not reinforcing in the presence of an alternative reinforcer [47].

It was recently revealed that JJC8-091 displayed significant binding affinity to the human *ether-à-go-go*-related gene (hERG) (IC_50_ = 2.32 ± 0.575 μM), as well as lower DAT affinity in nonhuman primates (*K*_i_ = 2.7 μM) [41, 46] and in HEK293 cells transfected with human DAT (hDAT) (*K*_i_ = 2.14 ± 0.11 μM) [22], compared to earlier binding data in rat brain tissue (*K*_i_ = 230 ± 40 μM) [38], collectively dampening enthusiasm for its further development. Of note, hERG affinity is considered a predictor of cardiovascular toxicity, as blockade of hERG may prolong the QT interval, potentially leading to the lethal cardiac arrhythmia called torsade de pointes [48, 49].

**Fig. 1:**
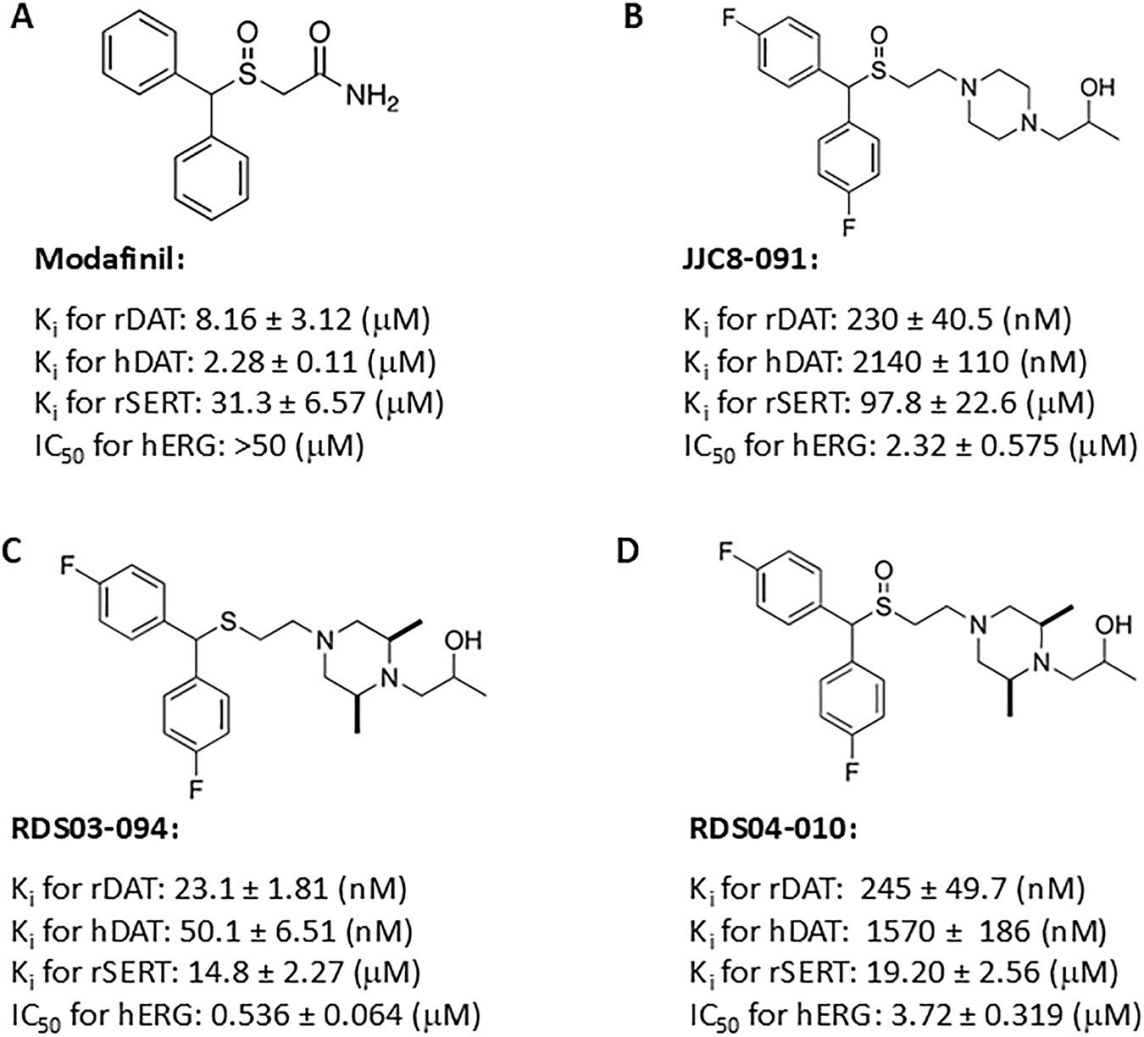
The chemicalstructures of modafinil and its analogs and their binding properties. **A** The chemical structure of modafinil and its binding properties on rat and human DAT (rDAT, hDAT), rat SERT (rSERT) and hERG. **B** The chemical structure of JJC8-091 and its binding properties on rDAT, hDAT, rSERT, and hERK. **C** The chemical structure of RDS03-094 and its binding on rDAT, hDAT, rSERT, and hERG. **D** The chemical structure of RDS04-010 and its binding properties on rDAT, hDAT, rSERT, and hERG.

In our ongoing efforts to improve the drug-like properties of these modafinil analogues we have attempted to improve binding affinities at hDAT vs. hERG, as well as increase metabolic stability [38, 41, 42]. We reported that the sulfide analogue, RDS-03-094, showed 10-fold higher DAT affinity (*K*_i_ = 23.1 nM) than JJC8-091 in rat brain tissue and a ~ 30-fold selectivity over hERG affinity [38, 41]. Preliminary behavioral testing suggested that RDS03-94 may have potential for development [38]. However, recent molecular dynamic simulations predicted that RDS-03-094 could be a cocaine-like DAT inhibitor, while its sulfoxide analog, RDS-04-010 (Fig. 1), was predicted to be an atypical DAT inhibitor [22]. In this study, we tested this hypothesis. We found that RDS-03-094 is indeed another typical DAT inhibitor with potential cocaine-like abuse potential, while RDS-04-010 is not cocaine-like and instead displays therapeutic potential with low addictive liability, as assessed in rodents.

## Materials and methods

### Animals

Male and female Long-Evans rats (Charles River Laboratories, Raleigh, NC, USA) were used for intravenous (i.v.) drug self-administration and reinstatement of drug seeking, and male and female DAT-cre mice with C57BL/6 J background (bred at the NIDA IRP Breeding Center, Baltimore, MD, USA) were used for optical intracranial self-stimulation (oICSS). All animals (rats and mice) were housed individually in a climate-controlled room under a 12 h light/dark cycle (lights on at 1900 h, lights off at 0700 h). Food and water were available *ad libitum* throughout the experiments. All experimental procedures were conducted in accordance with the Guide for the Care and Use of Laboratory Animals (National Research Council, 1996) and were approved by the Animal Care and Use Committee of the National Institute on Drug Abuse of the U.S. National Institutes of Health.

### Exp. 1: Cocaine self-administration and reinstatement tests in rats

Intravenous (i.v.) catheterization surgery and cocaine self-administration procedures were performed as described previously [50, 51]. After stable cocaine self-administration was achieved, the effects of RDS-03-094 (3, 10, 17 mg/kg) or RDS-04-010 (3, 10, 30 mg/kg, 30 min prior to testing) or vehicle (equal injection volume of 5% Kolliphor EL) on cocaine self-administration under fixed-ratio (FR2), progressive-ratio (PR), and multiple cocaine dose conditions were evaluated. In addition, the effects of RDS-03-094 (3, 10, 17 mg/kg) or RDS-04-010 (3, 10, 30 mg/kg) alone or its pretreatment on cocaine-induced reinstatement were also evaluated in separate groups of rats (see more details in the **S.I**.).

### Exp. 2: Oral sucrose self-administration in rats

Procedures for oral sucrose self-administration in mice were the same as we reported previously [50]. This experiment was designed to determine whether the same doses of RDS-03-094 (vehicle, 10, 17 mg/kg) or RDS-04-010 (vehicle, 10, 30 mg/kg) 30 min prior to testing inhibit non-drug reinforced behavior (see experimental details in the S.I.).

### Experiment 3: Open-field locomotion test

To determine whether the effects of both the RDS compounds on cocaine taking and seeking are due to non-specific sedation or locomotor impairment, we observed the effect of each compound alone on open-field locomotion in two additional groups of rats. The procedures for open-field locomotion testing were the following. Animals were given two consecutive daily sessions (1-hr per session) in the open-field chambers for habituation and minimization of novelty exploratory behavior. Then, on the following test days, animals were placed in the open-field chambers for 1-hr prior i.p. injections for baseline locomotion measurements. After baseline, the rats were injected with one of three doses (including the vehicle) of the drug and then immediately placed in the open-field apparatus to measure locomotion activity for 2-hr. The experiment was conducted in a within subject design with drug doses administered in a counterbalanced balance and at least 48-h between test days. To determine the percentage (%) change of baseline, only the last 30 min from the 1-hr baseline measurements (before i.p.) and the first 60 min from the 2-hr measurements (after i.p.) were included in the analysis.

### Experiment 4: Optogenetic brain-stimulation reward

To determine whether DA-dependent mechanisms underlie the effects of RDS-03-094 or RDS-04-010 on cocaine self-administration and reinstatement responding, we measured the effects of both compounds on optogenetic intracranial self-stimulation (oICSS) maintained by optical activation of ventral tegmental area (VTA) DA neurons in DAT-Cre mice expressing Cre-recombinase under the DA transporter (DAT) promoter. The optical ICSS procedures are the same as we reported previously [50, 52] (see details in the S.I.)

### Drugs

RDS-03-094 and RDS-04-010 were synthesized in the Medicinal Chemistry Section, NIDA-IRP by J. Cao, according to literature procedures [38]. They were dissolved in saline containing 10% DMSO and 15% tween-80 for intraperitoneal (i.p.) injection. In the substitution tests, the RDS compounds were dissolved in saline containing 2% DMSO and 4% tween-80. Cocaine HCl wase provided by NIDA IRP Pharmacy.

### Statistical analysis

All data are represented as the mean ± SEM. Animal group sizes were chosen based on a power analysis (*n* = 6–10 per group) and extensive previous experience with the animal models used. To validate the use of parametric statistics, we ensured that the residuals were normally distributed (Shapiro Wilk Test for normality; *p* > 0.05) and variances of the differences across all groups were equal (Levene’s test for homogeneity for between-subject ANOVA, *p* > 0.05 and Mauchly test for sphericity for repeated measures/mixed design ANOVA; *p* > 0.05). Statistical analysis was done using the independent values coming from individual animals in each group. One-way (or repeated measures, RM) ANOVA were utilized to analyze effects of different RDS-03-094 and RDS-04-010 doses on cocaine or sucrose self-administration under FR2 and PR schedules of reinforcement. Two-way repeated measures (RM) ANOVA was used to analyze the data in open-field locomotion, cocaine self-administration at multiple dose schedule, and oICSS. Two-way ANOVAs were utilized to analyze RDS compound- or cocaine-induced reinstatement tests. No data points were excluded from the analysis in any experiment. Where variation in group size occurred, this was due to animals being dropped from the experiment due to obstruction or clogging of i.v. catheters. Post hoc analyses were done using the Student-Newman-Keuls Method compared to vehicle/baseline control group. The value of *p* < 0.05 was used as the minimally acceptable statistically significant difference value in all tests. All tests were performed using SigmaStat 12.5 for Windows. The investigators were blinded to the group allocation during the experiments and data analysis.

## Results

### RDS-03-094 and RDS-04-010 are close analogs of JJC8-091

Figure 1 shows the chemical structures of modafinil, JJC8-091, RDS-03-094, and RDS-04-010, and their binding profiles at DAT and serotonin transporter (SERT) in rat brain tissue, illustrating that both the RDS compounds are highly selective DAT inhibitors (80–600-fold selectivity for DAT over SERT). In addition, hDAT and hERG binding profiles are included for comparison [22, 38, 41]. Notably, JJC8-091 and RDS-04-010 are both sulfoxides and display similar DAT binding profiles.

### RDS-03-094 and RDS-04-010 inhibit cocaine self-administration under FR2 reinforcement

**Fig. 2:**
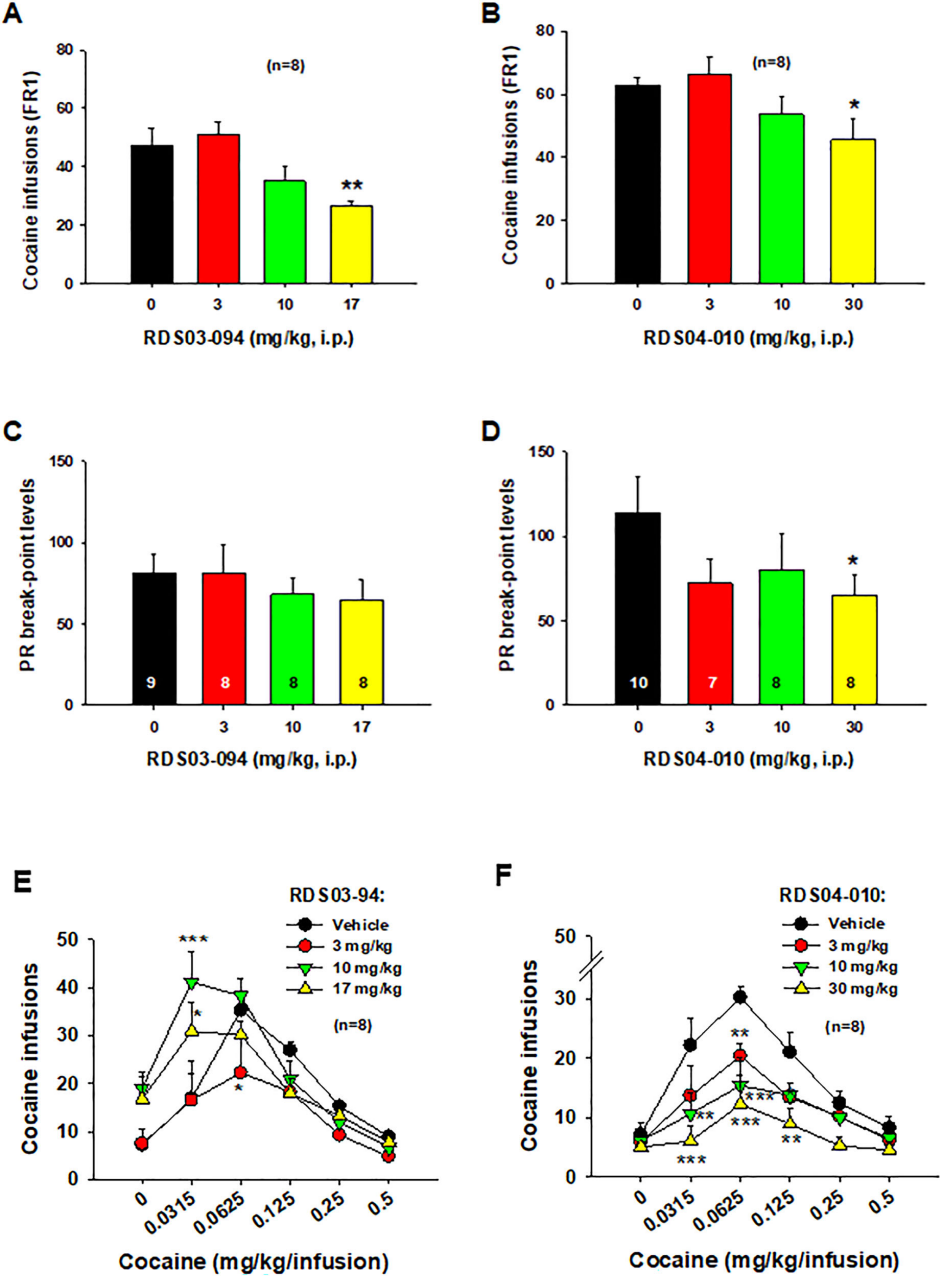
Effects of RDS-03-094 and RDS-04-010 on cocaine self-administration in rats. **A,** **B** Systemic administration of RDS-03-094 or RDS-04-010 dose-dependently decreased the number of cocaine infusions under a FR2 reinforcement schedule. **C** Systemic administration of RDS-03-094 failed to alter cocaine self-administration under progressive-ratio (PR) reinforcement schedule. **D** In contrast, RDS-04-010 significantly lowered the PR break-point level for cocaine self-administration at high doses. **E,** **F** Pretreatment with RDS-03-094 (**E**) shifted cocaine dose-response curve upward and to the left, while RDS-04-010 (**F**) produced a downward and rightward shift in the cocaine dose-response curve. *p < 0.05; **p < 0.01; ***p < 0.001, compared to the vehicle control group.

To determine whether both novel DAT inhibitors differentially alter drug-taking behavior, we first compared their effects on intravenous (i.v.) cocaine self-administration under an FR2 schedule of reinforcement. Figure 2 shows that RDS-03-094 dose-dependently decreased the number of cocaine infusions (Fig. 2A). Similarly, RDS-04-010 also dose-dependently decreased cocaine self-administration (Fig. 2B). One-way ANOVA for repeated measurement over drug dose revealed a significant treatment main effect (Fig. 2A: F_3,21_ = 7.24, *p* < 0.01; Fig. 2B: F_3,21_ = 3.86, *p* < 0.05). Post-hoc multiple group comparisons indicate a significant reduction after 17 mg/kg RDS-03-094 (**p < 0.01) or 30 mg/kg RDS-04-010 (*p < 0.05) when compared to the vehicle control group.

### RDS-04-010 inhibits cocaine self-administration under PR reinforcement schedule, while RDS-03-094 does not

We then evaluated the effects of these two compounds on break-point for cocaine self-administration under a progressive-ratio (PR) schedule of reinforcement, an index of motivation to seek drug. We found that RDS-03-094, at the same doses used above, failed to alter the PR break-point level (Fig. 2), while RDS-04-010 dose-dependently decreased the break-point for cocaine self-administration (Fig. 2D). One-way ANOVA failed to indicate a significant treatment main effect after RDS-03-094 (Fig. 2C: F_3,29_ = 0.45, *p* > 0.05), but revealed a significant treatment main effect after RDS-04-010 (Fig. 2D: F_3,28_ = 3.74, *p* < 0.05). Post-hoc multiple group comparisons revealed a significant reduction in break-point after 30 mg/kg RDS-04-010 when compared to the vehicle control (*p < 0.05).

### RDS-03-094 upward shifts, while RDS-04-010 downward shifts cocaine self-administration dose-response curve

We noted that the baseline levels of cocaine self-administration in the two groups of rats used to evaluate the effects of two RDS compounds (Fig. 2) differ slightly. This discrepancy is likely due to the use of separate groups with slight variations in age and testing periods. To address this concern, we directly compared the effects of both RDS compounds on multiple-dose cocaine self-administration in age-matched rats tested simultaneously (Fig. 2E, F). Systemic administration of RDS-03-094, at the doses of 10 and 17 mg/kg, significantly shifted the cocaine dose-response curve upward (Fig. 2E). In contrast, RDS-04-010 produced a dose-dependent reduction in cocaine self-administration and downward shifted the dose-response curve (Fig. 2F). Two-way RM ANOVAs indicate a significant cocaine dose main effect (Fig. 2E: F_5,35_ = 42.95, *p* < 0.001; Fig. 2F: F_5,35_ = 11.94, *p* < 0.001), drug treatment main effect (Fig. 2E: F_5,_ 35 = 2.51, *p* > 0.05; Fig. 2F: F_3,21_ = 7.73, *p* < .001), and cocaine dose × drug treatment interaction (Fig. 2E: F_15,105_ = 3.24, *p* < 0.01; Fig. 2F: F_15,105_ = 2.11, *p* < 0.05). Post-hoc individual group comparisons indicate a significant increase or decrease in cocaine self-administration after RDS-03-094 or RDS-04-010 administration. These findings align with our previous report, which showed that the atypical DAT inhibitor JJC8-091 significantly inhibited cocaine self-administration, whereas the typical DAT inhibitor JJC8-088 did not [29].

### RDS-03-094 and RDS-04-010 have no effect on oral sucrose self-administration in rats

**Fig. 3:**
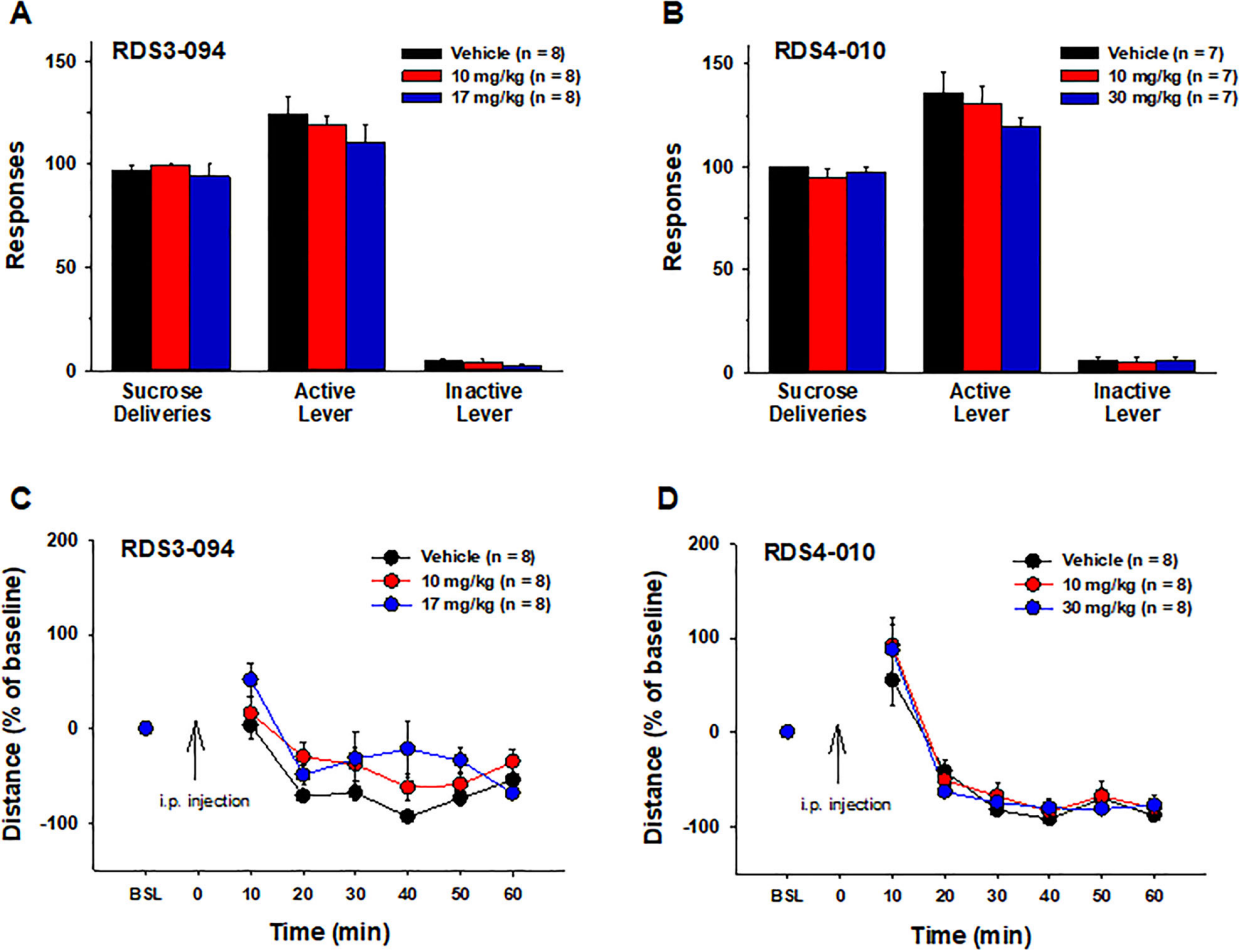
Effects of RDS-03-094 and RDS-04-010 on oral sucrose self-administration and open-field locomotion in rats. **A,** **B** Systemic administration of RDS-03-094 (10, 17 mg/kg) (**A**) or RDS-04-010 (10, 30 mg/kg) (**B**) failed to alter oral sucrose self-administration. **C,** **D** Similarly, systemic administration of RDS-03-094 (10, 17 mg/kg) (**C**) or RDS-04-010 (10, 30 mg/kg) (**D**) also failed to alter open-field locomotion.

To determine whether the actions observed above are cocaine-specific, we observed the effects of RDS-03-094 and RDS-04-010 on oral sucrose self-administration in rats. We found that systemic administration of the same doses that inhibit cocaine self-administration failed to alter oral sucrose self-administration (Fig. 3A, B). A one-way RM ANOVA did not reveal a significant RDS-03-094 treatment main effect (Fig. 3A, sucrose rewards, *F*_2,14_ = 0.513, *p* > 0.05; active lever responses, *F*_2,14_ = 1.436, *p* > 0.05). Similarly, a one-way RM ANOVA failed to reveal a significant RDS-04-010 treatment main effect (Fig. 3B, sucrose deliveries, *F*_2,12_ = 1.456, *p* > 0.05; active lever responses, *F*_2,12_ = 1.096, *p* > 0.05).

### RDS-03-094 and RDS-04-010 do not alter open-field locomotion

To further determine whether both the compounds produce sedative or locomotor impairment, and therefore, contributing to the reduction in cocaine self-administration, we observed their effects on open-field locomotion. Figure 3C shows that RDS-03-094 alone had no effect on locomotor activity. A two-way ANOVA failed to reveal either a RDS-03-094 treatment main effect (*F*_2,14_ = 2.322, *p* > 0.05) or time × dose interaction (*F*_12,84_ = 1.597, *p* > 0.05) although it revealed a significant time main effect (*F*_6, 42_ = 26.464, *p* < 0.001). Post-hoc group comparisons did not reveal significant differences in locomotion after either 10 mg/kg or 17 mg/kg RDS-03-094 administration compared to the vehicle control group (*p* > 0.05).

Similarly, RDS-04-010 alone failed to alter open-field locomotor activity. A one-way RM ANOVA failed to reveal a significant RDS-04-010 treatment main effect (Fig. 3D, *F*_2, 14_ = 0.447, *p* > 0.05) or time × dose interaction (*F*_12, 84_ = 0.665, *p* > 0.05) although it revealed a significant time main effect (*F*_6,42_ = 53.17, *p* < 0.001). Post-hoc group comparisons did not reveal significant differences in locomotion after either 10 mg/kg or 30 mg/kg RDS-03-094 administration as compared to the vehicle control group (*p* > 0.05).

### RDS-03-094 reinstates drug-seeking behavior, while RDS-04-010 does not

**Fig. 4:**
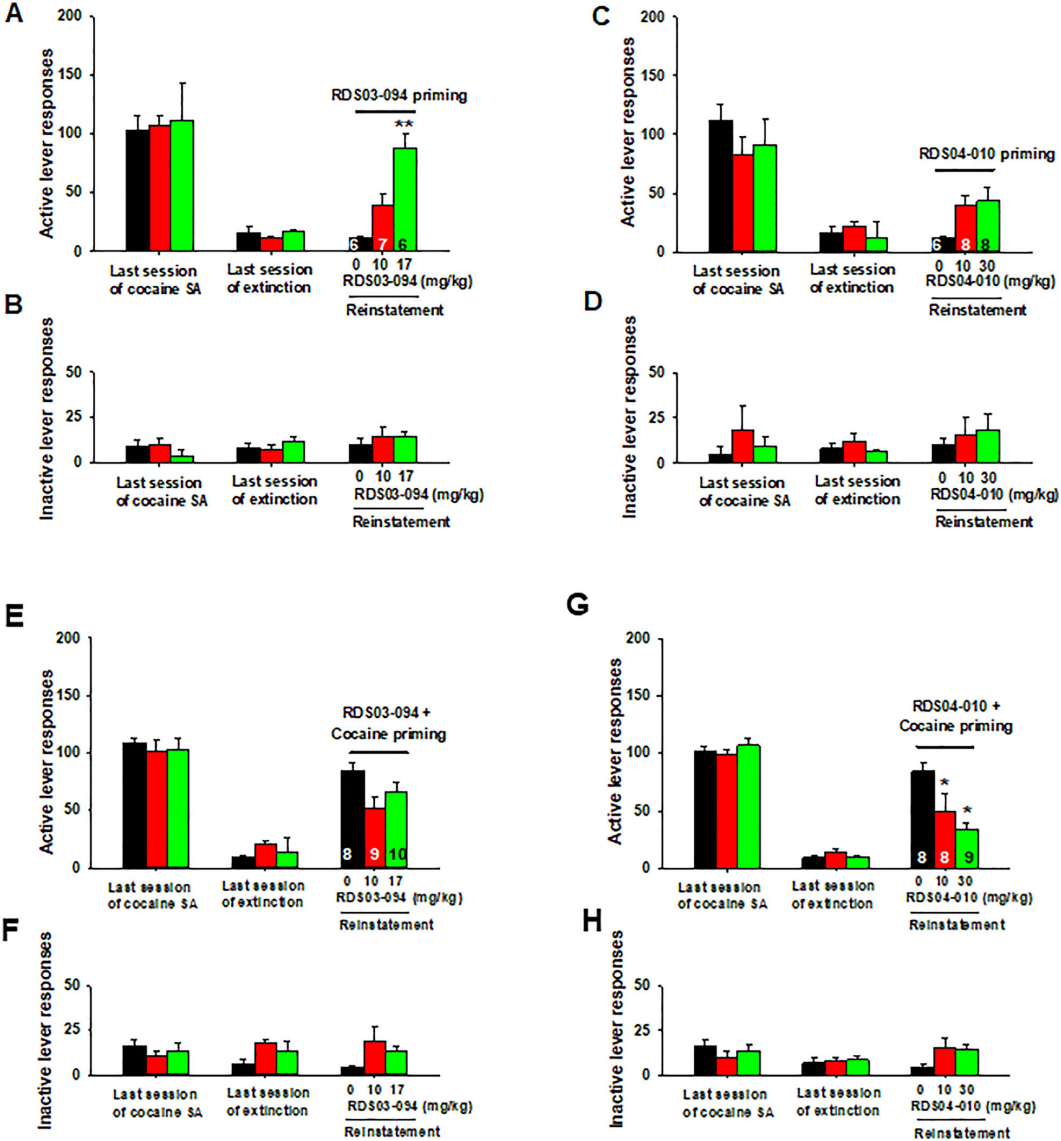
Reinstatement responding triggered by systemic (i.p.) administration of RDS-03-094 and RDS-04-010 in the absence or presence of cocaine in rats. **A**–**D** Systemic administration of RDS-03-094 (**A**) evoked significant reinstatement responding in rats after extinction from previous cocaine self-administration, while RDS-04-010 (**C**) did not. In contrast, neither of them altered inactive lever responding (**B**, **D**) compared to the vehicle (zero dose of drug) group. **E**–**H** Effects of RDS-03-094 or RDS-04-010 pretreatment on cocaine-primed reinstatement of drug-seeking behavior in rats. Cocaine (10 mg/kg, i.p.) priming produced a robust reinstatement responding in rats after extinction from previous cocaine self-administration (**E,** **G**, at 0 mg/kg test drugs). Pretreatment with RDS-03-094 (**E**) did not alter, while RDS-04-010 (**G**) significantly attenuated cocaine-triggered reinstatement of drug-seeking behavior. In contrast, cocaine priming did not alter inactive lever responding in the absence or presence of the RDS compound pretreatment (**F**), (**H**). *p < 0.05, **p < 0.01, com*p*ared to the vehicle (0 mg/kg RDS compound).

We then examined whether both RDS compounds produce similar reinstatement responses in rats whose responding was extinguished from previous cocaine self-administration. Figure 4 shows the total numbers of active and inactive lever responses observed during the last session of cocaine self-administration, the last session of extinction, and the reinstatement test session with different drug priming. RDS-03-094 priming (10, 17 mg/kg, i.p.) produced a robust and dose-dependent reinstatement response on the active lever (Fig. 4A), while RDS-04-010 priming at doses of 10 and 30 mg/kg did not evoke significant reinstatement response (Fig. 4C). Unlike active responses, neither drug priming altered inactive lever responses in the reinstatement test (Fig. 4B, D). An one-way ANOVA revealed a significant drug priming main effect after administration of RDS-03-094 (Fig. 4A: F_2,10_ = 40.49, *p* < 0.001), but not after RDS-04-010 (Fig. 4C: F_2,19_ = 3.17, *p* > 0.05). Post-hoc multiple group comparisons revealed a significant increase in reinstatement responding after 30 mg/kg RDS-03-094.

### RDS-04-010 pretreatment inhibits cocaine-induced reinstatement, while RDS-03-094 does not

We next examined whether pretreatment with either of the RDS compounds alters cocaine-induced reinstatement of drug-seeking behavior. Figure 4E shows that a single non-contingent cocaine-priming dose (10 mg/kg, i.p.) evoked robust reinstatement of cocaine-seeking behavior in rats whose lever responding was extinguished from previous cocaine self-administration in the absence of the RDS compound pretreatment. Pretreatment with RDS-03-094 (10, 17 mg/kg) failed to alter (Fig. 4E), while RDS-04-010 significantly attenuated cocaine-triggered reinstatement of drug-seeking behavior in rats in a dose-dependent manner (Fig. 4G). An one-way ANOVA did not reveal a significant pretreatment main effect after administration of RDS-03-094 (Fig. 4E, F_2,24_ = 3.37, *p* > 0.05), but revealed a significant RDS-04-010 treatment main effect (Fig. 4G, F_2,22_ = 5.57, *p* < 0.05). Post-hoc multiple group comparisons revealed a significant reduction in cocaine-triggered reinstatement after 10 mg/kg or 30 mg/kg RDS-04-010 (Fig. 4G). There is no significance change in inactive lever responses after RDS-03-094 (Fig. 4F) or RDS-04-010 (4H) administration.

### RDS-03-094 enhances optical brain-stimulation reward, while RDS-04-010 does not

**Fig. 5:**
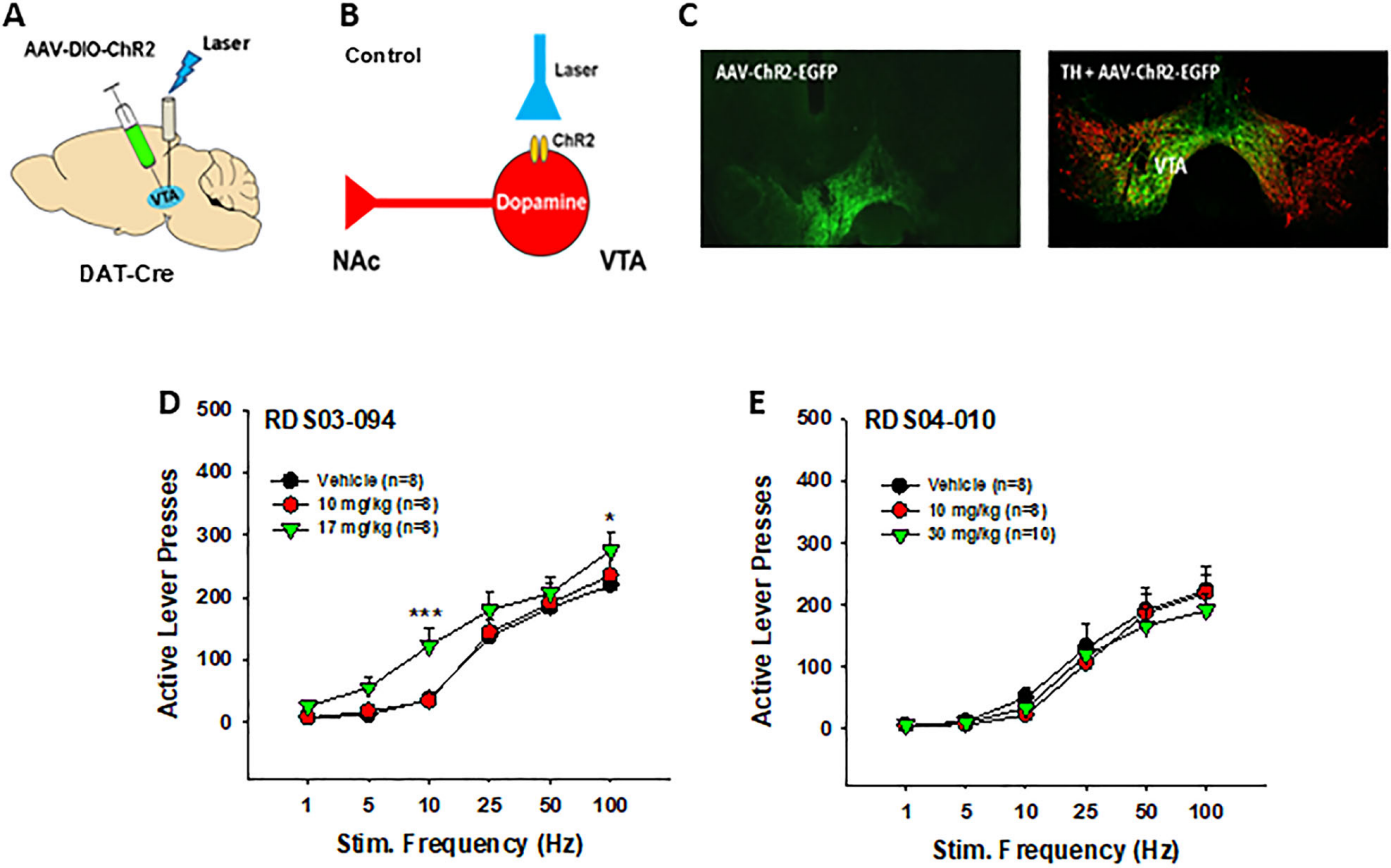
Effects of RDS-03-094 and RDS-04-010 on optical intracranial self-stimulation (oICSS) in DAT-cre mice. **A** A diagram showing the experimental methods for oICSS. AAV-ChR2-eYFP viruses were microinjected into the VTA of DAT-cre mice and optical fibers were implanted into the VTA to optically excite VTA DA neurons contingently upon lever response. **B** A diagram showing that AAV-ChR2-eGFP is selectively expressed in VTA DA neurons and contingent active lever pressing leads to laser delivery that subsequently activate VTA DA neurons. **C** Representative images of AAV-ChR2-EYFP expression (green), illustrating EGFP co-localization with TH (red) in VTA DA neurons. **D** Systemic administration of RDS-03-094 shifted the frequency – rate response curve leftward or upward. **E** RDS-04-010 had no effect on oICSS, suggesting that it is not rewarding or aversive. *p < 0.05, ***p < 0.001, com*p*ared with the vehicle control group.

To examine whether either RDS compound alters brain reward function, we expressed light-sensitive channelrhodopsin 2 (ChR2) into VTA DA neurons in DAT-cre mice (Fig. 5A–C), then observed the effects of both the compounds on optical intracranial self-stimulation (oICSS). Figure 5D shows the mean rate-frequency function curves, illustrating a typical sigmoidal-shape curve after vehicle treatment in DAT-cre mice. Pretreatment with RDS-03-094 dose-dependently shifted the stimulation frequency–rate response curve upward (Fig. 5D), suggesting that RDS-03-094 produces an enhancement in oICSS, similar to cocaine or JJC8-088 [29]. In contrast, pretreatment with RDS-04-010 failed to alter the rate-frequency function curve (Fig. 5E), suggesting a non-significant effect on DA-dependent oICSS behavior by itself. This is different from JJC8-091 that inhibits oICSS by itself under the same experimental conditions [29]. A two-way RM ANOVA revealed a significant stimulation frequency main effect (Fig. 5D, F_5,35_ = 52.12, *p* < 0.001), RDS-03-094 treatment main effect (F_2,14_ = 5.27, *p* < 0.05), and frequency × treatment interaction (F_10,70_ = 2.48, *p* < 0.05). The same two-way RM ANOVA for the data shown in Fig. 5E revealed a significant stimulation frequency main effect only (F_5,35_ = 34.45, *p* < 0.001), but didn’t reveal RDS-04-010 treatment main effect (F_2,14_ = 1.74, *p* > 0.05) or frequency × treatment interaction (F_10,70_ = 1.71, *p* > 0.01). Post-hoc individual group comparisons revealed a significant increase in oICSS (e.g., active lever response) after 30 mg/kg RDS-03-094 at 10 and 100 Hz.

### RDS-03-094 can maintain self-administration in cocaine substitution test, while RDS-04-010 does not

**Fig. 6:**
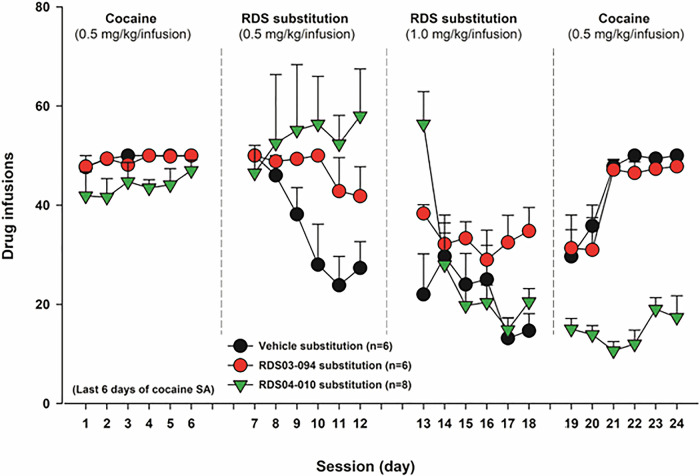
Drug substitution tests in cocaine self-administration rats. Naïve rats were initially trained for cocaine self-administration until the stable self-administration was achieved for at least 5 days. Then the animals were divided into 3 drug substitution groups (saline, RDS-03-094, RDS-04-010). RDS-03-094 substitution (initially 0.5 mg/kg/infusion for 5 days followed by 1.0 mg/kg/infusion for additional 5 days) sustained slightly lower rate, but stable self-administration in rats previously self-administered cocaine. When RDS-03-094 was replaced with cocaine again, the animals rapidly re-acquired stable self-administration. In contrast, RDS-04-010, at the dose of 0.5 mg/kg/infusion, appeared to maintain self-administration. However, at a higher dose (1.0 mg/kg/infusion), RDS-04-010 failed to maintain self-administration. The animals quickly quit self-administration in a way similar to saline substitution. When RDS-04-010 was replaced with cocaine again, the animals did not show cocaine-like self-administration behavior.

Finally, we examined whether RDS-03-094 or RDS-04-010 is cocaine-like in its reinforcing effects, by examining whether the RDS compounds are able to maintain self-administration in a cocaine substitution test. After stable self-administration was achieved, cocaine was replaced by vehicle, RDS-03-094, or RDS-04-010. When cocaine was replaced by RDS-03-094 (initially 0.5 mg/kg/infusion for 5 days, followed by 1.0 mg/kg/infusion for additional 5 days) in one group of rats, RDS-03-094 substitution maintained stable self-administration in a dose-dependent manner (Fig. 6). In contrast, RDS-04-010 substitution produced mixed effects depending on the drug doses. At a low dose (0.5 mg/kg/infusion), RDS-04-010 appeared to be able to maintain self-administration as assessed by averaged active responses that are comparable to those in the cocaine self-administration phase (Fig. 6). However, when the dose of RDS-04-010 was increased to 1 mg/kg/infusion, animals gradually quit self-administration during 5 days of substitution tests in a way similar to saline-induced extinction. Notably, when the test drug was replaced with cocaine, the rats tested with RDS-03-094 rapidly re-acquired self-administration behavior, while the rats tested with RDS-04-010 did not. These findings suggest that chronic RDS-04-010 administration during 10 days of the substitution test produced a prolonged inhibitory effect on cocaine self-administration, while RDS-03-094 did not. Together, these findings suggest that RDS-03-094 is reinforcing like cocaine, while RDS-04-010 is not.

## Discussion

This study presents two major findings. First, as predicted by computational modeling [22], the novel compound RDS-03-094 appears to behave as a typical DAT inhibitor. Similar to other typical DAT inhibitors such as JJC8-088 [29], RDS-03-094 enhanced cocaine self-administration in multiple-dose tests, increased oICSS, induced reinstatement of drug-seeking, and sustained self-administration when substituted for cocaine. These findings suggest that RDS-03-094 may have low or limited therapeutic potential for CUD. Second, RDS-04-010 is identified as an atypical DAT inhibitor. Unlike typical DAT inhibitors, RDS-04-010 itself did not exhibit reinforcing potential, as indicated by its lack of effect in oICSS, reinstatement, and drug substitution tests. Pretreatment with RDS-04-010 inhibited cocaine self-administration under multiple reinforcement conditions and attenuated cocaine-triggered reinstatement of drug-seeking behavior. Furthermore, neither compound affected oral sucrose self-administration nor open-field locomotor activity. These findings suggest that the rats were not impaired, and their behavioral response to cocaine differed from a natural reinforcer. Unexpectedly, RDS-03-094 failed to produce a cocaine-like locomotor effect given that it is a typical DAT inhibitor. This result aligned with our previous observation in mice, where RDS-03-094 (corresponding to compound 14a) at a wide dose range (1, 3, 10, 30 mg/kg) also failed to produce cocaine-like hyperactivity [38]. Together, these findings highlight the translational potential of RDS-04-010, but not RDS-03-094, for the treatment of CUD.

We have previously reported on two similar DAT inhibitors, JJC8-088 and JJC8-091, which differ subtly in structure (e.g., the presence of a terminal phenyl group in JJC8-088, which is absent in JJC8-091) [28], yet exhibit distinct behavioral profiles in experimental animals [29]. JJC8-088 demonstrated a cocaine-like behavioral profile, in self-administration, oICSS, and reinstatement and substitution tests, while JJC8-091 did not in any of these behavioral models [29]. Importantly, pretreatment with JJC8-091 inhibited cocaine self-administration and reinstatement of drug-seeking behavior [29], suggesting that JJC8-091 may have therapeutic potential for treatment of psychostimulant use disorders. This was supported by the finding that JJC8-091 also dose-dependently inhibited short-access, and particularly long-access, methamphetamine self-administration in rats [45]. However, in a subsequent study in non-human primates both JJC8-088 and JJC8-091 exhibited modest effects in reducing the choice for cocaine over food rewards in a pilot study (*n* = 3 rhesus monkeys) after chronic administration [46]. Additionally, JJC8-091 showed lower binding affinity for DAT in both rhesus monkey brain tissue and in hDAT transfected HEK293 cells [41, 46] (Fig. 1) and comparable hERG channel affinity. The latter finding may predict cardiotoxicity. Although JJC8-091 was not reinforcing in the presence of an alternative reinforcer in cocaine-experienced rhesus monkeys [47], the data did not support further development of this drug.

Both RDS-03-094 and RDS-04-010 are analogs of JJC8-091, featuring either a sulfide moiety (RDS-03-094) or a sulfoxide moiety (RDS-04-010) (Fig. 1). Initially, we designed and synthesized these analogues to improve DAT affinity, metabolic stability and reduce hERG activity [38]. Of note, JJC8-089, the sulfide analogue of JJC8-091 proved to be highly metabolically unstable mitigating further behavioral investigations. The 2,6-dimethyl substitution on the piperazine ring was incorporated to improve metabolic stability while retaining DAT affinity (Fig. 1) [38].

While preliminary data suggested that RDS-03-094 might be a lead compound for treating CUD [38], extensive quantum mechanical calculations and molecular dynamics simulations revealed that the sulfoxide moiety of JJC8-091 and RDS-04-010 is critical for binding the inward facing conformation of DAT. RDS-03-094 prefers to bind to an outward-facing conformation of DAT, predicting a cocaine-like behavioral profile, whereas its sulfoxide analogue, RDS-04-010, preferentially binds to an inward-facing conformation [22]. This suggested that RDS-04-010 might act as an atypical DAT inhibitor [22]. Our results from a series of behavioral assays indeed support this prediction. Notably, the only structural difference between RDS-03-094 and RDS-04-010 is the presence of a sulfide group in RDS-03-094 and a sulfoxide group in RDS-04-010. This subtle difference appears to determine their binding preference on DAT and functional activity in vivo.

In summary, in this study, we compared the behavioral pharmacological effects of both RDS-03-094 and RDS-04-010, a pair of novel typical and atypical DAT inhibitors, in animal models of addiction. We found that RDS-03-094 is a typical DAT inhibitor, producing robust cocaine-like behaviors in multiple-dose cocaine self-administration, brain-stimulation reward and reinstatement tests. In contrast, RDS-04-010 was identified as another atypical DAT inhibitor, which does not produce cocaine-like behavior in any of these models. Moreover, pretreatment with RDS-04-010 inhibits cocaine taking and seeking, while itself is not rewarding or aversive. Given that RDS-04-010 has similar binding affinity to DAT and hERG activity as JJC8-091 [41], further research is needed to determine the development potential of RDS-04-010 in treating CUD. Significant progress in recent DAT-based medication development will undoubtedly be inspired by the recent cryoEM structures that show structurally different DAT inhibitors binding in a unique fashion, complimenting 30 years of drug design and development [17–19]. These structures as well as novel drug design that includes a series of very interesting thiazole [53–56] and biphenyl analogues of modafinil [41] hold promise for development towards therapeutics for CUD as well as cognitive disorders associated with aging and substance use disorders.

## Supplementary information

Supplementary Materials

## Data Availability

The data that support the findings of this study are available from the corresponding authors upon request.
