# Supplementary material for: RDS-04-010: a novel atypical DAT inhibitor that inhibits cocaine taking and seeking and itself has low abuse potential in experimental animals
Source: Transl Psychiatry. 2025 May 24;15:182. doi: 10.1038/s41398-025-03391-7 (PMC12103503; doi:10.1038/s41398-025-03391-7)
Supplement: Supplementary file 1 — Supplementary Materials [file 41398_2025_3391_MOESM1_ESM.docx]

Translational Psychiatry

**RDS-04-010: A novel atypical DAT inhibitor that inhibits cocaine taking and seeking and itself has low abuse potential in experimental animals**

Omar Soler-Cedeno^1,^ *, Ewa Galaj ^1,2,^*, Benjamin Klein^1^, Jianjing Cao^1^, Guo-Hua Bi^1^, Amy Hauck Newman^1, #^, Zheng-Xiong Xi^1, #^

**Supplementary information**

**Materials and methods**

**Exp. 1: Intravenous drug self-administration**

***Surgery:*** Rats used in cocaine self-administration experiments were implanted i.v. with a microrenathane catheter (Braintree Scientific Inc., Braintree, MA, USA). Each rat was anaesthetized first with a mixture of ketamine (100 mg/kg) and xylazine (10 mg/kg, i.p.) and then a small incision was made to the right of the midline of the neck to expose the external jugular vein. One end of the i.v. catheter was next inserted into the vein with the catheter tip reaching the right atrium. The catheter was then secured to the vein with silk suture and the other end fed subcutaneously around the back of the neck to exit near the back of the skull, connected to a bent 24-gauge stainless steel cannula (Plastics One Inc., Roanoke, VA, USA). The catheter and the guide cannula were secured to the skull with four stainless steel screws threaded into the skull and dental cement. The incision was then sutured.

***Cocaine self-administration training***: Drug self-administration experiments were conducted in operant response test chambers from Med Associates Inc. (Georgia, VT, USA). Each test chamber had an active lever and an inactive lever. Depression of the active lever activated the infusion pump; depression of the inactive lever was counted but had no consequence. After 5-7 days of recovery from surgery, rats were initially trained to self-administer cocaine (1.0 mg/kg/infusion) under FR1 reinforcement. Each cocaine infusion delivered a volume of 0.08 mL/infusion over 4.6 s and was paired with presentation of a stimulus light and tone. During the 4.6 s infusion time, additional responses on the active lever were recorded but did not lead to additional infusions. Each session lasted 3 h. FR1 reinforcement was used for 5-7 days. Then subjects were allowed to continue cocaine (0.5 mg/kg/infusion) self-administration under FR2 reinforcement until stable cocaine self-administration was established: a minimum of 20 presses on the active lever per test session and stability criteria of less than 10% variability in inter-response interval, less than 10% variability in number of infusions taken, and less than 10% variability in number of presses on the active lever for at least 3 consecutive days. The dose of cocaine was chosen based on previous studies showing that 0.5 mg/kg/infusion of cocaine lies within the middle range of the descending limb of the cocaine dose-response self-administration curve, where reliable dose-dependent effects can be observed ^50, 51^. In addition, we selected 0.5 mg/kg of cocaine, rather than 1 mg/kg, to increase the work demand (i.e., lever presses) on the rats while maintaining the same drug intake. In our experience, this approach increases the sensitivity of measuring changes in drug-taking and drug-seeking behavior. To avoid cocaine overdose during the self-administration period, each animal was limited to a maximum of 50 cocaine injections per 3 h session.

***Effects of RDS-03-094 and RDS-04-010 on FR2 cocaine self-administration:*** The effects of RDS-03-094 (3, 10, 17 mg/kg) or RDS-04-010 (3, 10, 30 mg/kg) 30 min prior to testing, on cocaine self-administration were evaluated after stable cocaine self-administration was established for at least 3 consecutive days. After each test, rats then received an additional 3-5 days of self-administration of cocaine alone until stable self-administration was re-established. The order of testing for the various doses of the compound was counterbalanced.

***Effects of RDS-03-094 and RDS-04-010 on PR cocaine self-administration****:* The initial cocaine self-administration training under FR1 and FR2 reinforcement schedules was identical to that outlined above. After stable cocaine self-administration (1.0 mg/kg/infusion) under FR2 reinforcement was established, the subjects were switched to cocaine self-administration (0.5 mg/kg/infusion) under a PR schedule, during which the work requirement of lever presses needed to receive a single i.v. cocaine infusion was progressively raised within each test session according to the following PR series: 1, 2, 4, 6, 9, 12, 15, 20, 25, 32, 40, 50, 62, 77, 95, 118, 145, 178, 219, 268, 328, 402, 492 and 603 until the break point was reached ^52^. The break-point was defined as the maximal workload (i.e. number of lever presses) completed for the last cocaine infusion prior to a 1-h period during which no infusions were obtained by the animal. Animals were allowed to continue daily sessions of cocaine self-administration under PR reinforcement conditions until day-to-day variability in break point fell within 1–2 ratio increments for 3 consecutive days. Once a stable break-point was established, subjects were assigned to two subgroups to determine the effects of RDS-03-094 (3, 10, 17 mg/kg), RDS-4-010 (3, 10, 30 mg/kg, i.p.) or vehicle (1 mL/kg of sterile water containing 10% DMSO and 15% Tween-80) on PR break-point for cocaine self-administration.

***Effects of RDS-03-094 and RDS-04-010 on multiple-dose cocaine self-administration:***To further explore the pharmacological efficacy of these novel compounds, we assessed whether they reduce cocaine self-administration maintained by a full range of cocaine doses. Within each session, rats self-administered multiple cocaine doses (0, 0.0315, 0.0625, 0.125, 0.25, and 0.5 mg/kg/infusion) every 20 min in a descending dose sequence under a FR2 schedule of reinforcement. Cocaine concentration was adjusted by changes in the infusion volumes and duration of pump activation. After stable cocaine self-administration was achieved as defined above, animals (n=8) received a systemic administration of RDS-03-094 (3, 10, 17 mg/kg) or RDS-04-010 (3, 10, 30 mg/kg) 30 min prior to the test session and later were allowed to self-administer the different doses of cocaine under the same conditions. Animals were tested with different doses of the drugs once their self-administration baselines were re-established. Tests were conducted 3-5 days apart. The order of testing with different doses of the drug was counterbalanced.

***Drug substitution test in cocaine self-administration rats:*** Three additional groups of rats were used to evaluate the addictive liability of RDS-03-094, RDS-04-010, or vehicle. Animals were initially trained for cocaine self-administration with the procedures as described above, followed by vehicle, RDS-03-094 or JJC8-091 substitution, respectively, with the same doses of cocaine. After stable cocaine self-administration was established for at least 5 consecutive days, the cocaine self-administration rats were switched to self-administer RDS-03-094 or RDS-04-010 (0.5 mg/kg/infusion) for 5 days, followed by 1.0 mg/kg/infusion for additional 5 days). Since rats might take several days to support self-administration for a novel reinforcer, each replacement test was continued for 10 days. After the 10 days of substitution, animals were re-exposed for cocaine (0.5 mg/kg/infusion) for 3-5 days to determine whether the drug substitution alters cocaine self-administration.

***Reinstatement of drug-seeking behavior:*** After stable cocaine self-administration was established, rats underwent response extinction. The extinction procedures were the same as described previously ^51^. During extinction, cocaine was replaced by saline, and the cocaine-associated cue-light and tone were turned off. Active lever-pressing led only to saline infusion. After the rats met the extinction criterion (≤10 lever presses for 3 consecutive days), they were divided into eight drug (dose) groups to study the effects of each dose of RDS-03-094 (10, 17 mg/kg, i.p.), RDS-04-010 (3, 10 mg/kg, i.p), or the vehicle (n=8-11 per dose, between-subjects design) alone on reinstatement of drug-seeking behavior, respectively. Additional eight groups of rats were used to study the effects of RDS-03-094, or RDS-04-010 pretreatment (at the same doses as stated above, 30 min prior to testing) on 10 mg/kg cocaine-induced reinstatement of drug-seeking behavior.

**Experiment 2: Oral sucrose self-administration in rats**

Two additional groups of rats were trained to self-administer sucrose under an FR1 schedule of reinforcement during daily 2-hr sessions. Responding on the active lever activated the syringe pump causing the delivery of 5% liquid sucrose onto a liquid food receptacle (0.01 mL per delivery) and the presentation of the light/tone cue above the active lever. Responses on the inactive lever were counted but had no consequences. During the pump activation/infusion period (4.6 s, Med Associates, Model PHM-100), additional responses on the active lever were recorded but did not lead to additional sucrose deliveries. To prevent satiation of sucrose reward, we set a maximal number of 100 sucrose deliveries during each 2-hr session. After stable sucrose self-administration was achieved, defined as (i) at least 20 sucrose rewards earned per 2-hr session, (ii) less than 20% variability in daily sucrose intake across two consecutive sessions, and (iii) an active/inactive lever press ratio exceeding 2:1, the rats randomly received vehicle or one of two doses of RDS-03-094 (10 or 17 mg/kg, i.p.) or RDS-04-010 (10 or 30 mg/kg, i.p.). The treatment was counterbalanced in each rat, and each test was separated by 2-4 additional training sessions. The total number of sucrose deliveries during the 2-hr self-administration session and the numbers of active and inactive lever responses were used to evaluate the effects of the test compounds on oral sucrose self-administration. The experiment was conducted in a within-subjects design.

**Experiment 4: Optical brain-stimulation reward**

***Surgery***: DAT-cre mice (~4 weeks of age) were anesthetized with ketamine (90 mg/kg, i.p.) and xylazine (10 mg/kg, i.p.) and placed in a stereotaxic frame (David Kopf Instruments, Tujunga, CA, USA). For intra-VTA microinjection of virus, a custom-made 30-gauge stainless injector was used to infuse Cre-inducible recombinant adeno-associated virus (AAV) that encodes channelrhodopsin-2 (ChR2) and enhanced green fluorescent protein (EGFP) (i.e., AAV- EF1α-DIO-ChR2-EGFP) or the control virus (AAV2-EF1α-DIO-EGFP) (300 nL, ∼2 × 1012 genomes/mL, University of North Carolina Gene Therapy Center) unilaterally into the VTA (AP -3.2; ML 0.1; DV -4.2 mm relative to Bregma) using a micropump (WPI 2000 UltraMicroPump, Sarasota, FL, USA) with a speed of 50 nL/min. For optical brain stimulation, a custom-built optrode (200-μm multimode optical fiber, Thorlabs, Newton, NJ, USA) tethered to an intracranial ceramic ferrule (MMFER2007C-2300, Precision Fiber Products, Inc., Milpitas, CA, USA) was implanted into the VTA (AP −0.32; ML 0.1, DV −3.7 mm relative to Bregma) at the AAV injection site. Dental cement was used to fix the optrode assembly to the skull. Following AAV vector injection and optrode implantation, mice were allowed to recover for at least 2 weeks before optical self-stimulation experiments began.

***Optical intracranial self-stimulation (oICSS) device****:* Optical stimulation experiments were conducted in standard operant conditioning chambers (Med Associates, Fairfax, VT, USA). Each chamber was equipped with two wall-mounted levers, two cue lamps, a house lamp, an audio stimulus generator, and four pairs of infrared detectors. Mice were gently connected to a cable that was in turn connected to a 473 nm laser tuned for ChR2 stimulation via an optical swivel. Computer software controlled a pulse generator that controlled the lasers.

***oICSS Procedure:*** The general procedures for oICSS were the same as we reported previously ^54^. After 4 weeks of recovery from surgery, mice were placed into operant chambers containing two operant levers – an active lever and an inactive lever, respectively (ENV-307W-CT, Med associates Inc., Fairfax, VT, USA). The optrode implanted into the mouse brain (VTA) was connected to a 473 nm laser (OEM Laser Systems, Inc., Draper, UT, USA) via an optical swivel (Doric Lenses Inc, Quebec, Canada). Animals were initially trained on a fixed-ratio 1 (FR1) reinforcement schedule; each active lever response led to delivery of a 1-s pulse train of light stimulation (473 nm, 20 mW, 5 ms duration, 25 Hz) accompanied by a 1-s illumination of cue light above the lever. While inactive lever presses were counted, they had no programmed consequence. Each daily training session lasted 60 min. An additional group of mice received a lever switch test in which the assignment of active and inactive levers with respect to the right and left levers was reversed to confirm that the lever responding was photostimulation reward-contingent. Following establishment of lever-pressing for oICSS, animals were presented with a series of 6 different stimulation frequencies (100, 50, 25, 10, 5, 1 Hz) in descending order to obtain rate-frequency response curves. Animals were allowed to respond for 10 min per stimulation frequency. The animals were then divided into 2 groups (6 mice per group) to observe the effects of RDS-03-094 (10, 17 mg/kg), RDS-04-010 (10, 30 mg/kg, i.p.), or vehicle, respectively, on optical BSR maintained by photostimulation of VTA DA neurons in DAT-Cre mice. Each animal received 3 drug injections during the oICSS experiments. After each test, animals received an additional 5-7 days of oICSS re-stabilization until a new baseline of lever responding was established. The order of testing for the various doses of the drugs was counterbalanced. The effects of the RDS compounds on oICSS were evaluated by comparing drug-induced changes in active lever presses in DAT-Cre mice.
